# BRCA1-Associated Protein Is a Potential Prognostic Biomarker and Is Correlated With Immune Infiltration in Liver Hepatocellular Carcinoma: A Pan-Cancer Analysis

**DOI:** 10.3389/fmolb.2020.573619

**Published:** 2020-11-02

**Authors:** Qiang Ju, Xin-mei Li, Heng Zhang, Yan-jie Zhao

**Affiliations:** ^1^Department of Blood Transfusion, The Affiliated Hospital of Qingdao University, Qingdao University, Qingdao, China; ^2^School of Public Health, Qingdao University, Qingdao, China

**Keywords:** BRAP, tumorigenesis, prognosis, immune infiltrate, pan-cancer

## Abstract

**Background:**

BRCA1-associated protein (BRAP) is a critical gene that regulates inflammation-related signaling pathway and affects patients’ prognosis in esophageal squamous cell carcinoma (ESCC). However, its roles in different cancers remain largely unknown.

**Methods:**

BRAP expression in human pan-cancer was analyzed *via* the Genotype-Tissue Expression (GTEx) and The Cancer Genome Atlas (TCGA) database. Pearson correlation analysis was used to evaluate the association between BRAP expression with mismatch repair (MMR) gene mutation and DNA methyltransferase. We evaluated the influence of BRAP on clinical prognosis by univariate survival analysis. Moreover, the correlation between BRAP and tumor immune infiltration was analyzed *via* the Tumor Immune Evaluation Resource (TIMER) database. Pearson correlation analysis was used to investigate the correlation between BRAP expression and immune checkpoint genes expression.

**Results:**

BRAP is abnormally overexpressed and significantly correlated with MMR gene mutation level and DNA methyltransferase expression in human pan-cancer. Univariate survival analysis showed that BRAP was significant with patients’ overall survival (OS) in six cancer types, disease-free interval (DFI) in three cancer types, and progression-free interval (PFI) in two cancer types. Remarkably, increased BRAP expression was strongly correlated with patients’ poor prognosis in liver hepatocellular carcinoma (LIHC), whether OS (*P* < 0.0001, hazard ratio (HR) = 1.1), DFI (*P* = 0.00099, HR = 1.06), or PFI (*P* = 0.00025, HR = 1.07). Moreover, a positive relationship was found between BRAP expression and immune infiltrating cells including B cell, CD4 + T cell, CD8 + T cell, dendritic cell, macrophage cell, and neutrophil cell in colon adenocarcinoma (COAD), kidney renal clear cell carcinoma (KIRC), and LIHC. Additionally, BRAP expression showed strong correlations with immune checkpoint genes in LIHC.

**Conclusion:**

BRAP expression is increased in human pan-cancer samples compared with normal tissues. Overexpression of BRAP is correlated with poor prognosis and immune infiltration in multiple cancers, especially in LIHC. These findings suggest that BRAP may be used as a potential molecular biomarker for determining prognosis and immune infiltration in LIHC.

## Introduction

BRCA1-associated protein (BRAP) was first reported to bind to breast cancer suppressor protein BRCA1. It functions as an E3 ubiquitin ligase and could modulate mitogen-activated protein kinase (MAPK) signaling pathway (Matheny et al., 2004). In our previous two studies, we found that BRAP was an esophageal squamous cell carcinoma (ESCC) susceptible gene (Wu et al., 2011) and played an important role in ESCC invasion and metastasis (Zhao et al., 2017). Copy number gain caused BRAP overexpression to activate nuclear factor (NF)-κB signaling pathway and affected patients’ survival (Zhao et al., 2017). Although the function of BRAP in ESCC is very clear, its roles in human pan-cancer are still largely unknown.

Tumor immunology is a trending topic in cancer research. Immune-related mechanisms play important roles in multiple pathophysiological progresses (Rožman, 2018; Gonzalo and Coll-Bonfill, 2019; Walker et al., 2019), and immunotherapeutic strategies are considered as a promising direction for the treatment of many cancers (Washah et al., 2020). Immunotherapy, such as programmed death-1 (PD-1) and programmed death ligand-1 (PD-L1) inhibitors, plays a good anti-tumor effect in the treatment of malignant tumors, such as lung cancer (Cheng et al., 2020; Eguren-Santamaria et al., 2020) and melanoma (Cuevas and Daud, 2018; Albershardt et al., 2020). Moreover, increasing numbers of studies showed that tumor-infiltrating lymphocytes, such as tumor-associated macrophages (TAMs) and tumor-infiltrating neutrophils (TINs), were directly related to the patients’ prognosis and efficacy of immunotherapy (Bocchialini et al., 2020; He et al., 2020; Schirosi et al., 2020; Stenzel et al., 2020). Therefore, the study of the interaction between tumor and immunity and identification of novel targets of immunotherapy are of great clinical significance for the treatment of patients. Previous study showed that BRAP expression can be induced by inflammatory stimulation and activates inflammatory cascades in carotid atherosclerosis (Liao et al., 2011). However, the underlying mechanism of BRAP in tumor immunology is still unclear.

In the present study, we comprehensively analyzed the association between BRAP expression and patients’ prognosis in 33 cancer types. Moreover, we explored the correlation of BRAP expression with 6 tumor-infiltrating immune cells and immune checkpoint in 33 tumor microenvironments. Our findings revealed the possible role of BRAP across cancers, suggesting that BRAP is a potential prognostic biomarker and is correlated with immune infiltration in many cancers, especially in LIHC.

## Materials and Methods

### Sample Information and BRAP Expression Analysis in Human Pan-Cancer

BRAP expression data of 31 normal tissues (liver, lung, kidney, brain, bone marrow, etc.) were obtained from the Genotype-Tissue Expression (GTEx) program and downloaded through the GTEx portal^1^. BRAP expression data of 21 tumor cell lines were obtained from the CCLE database^2^. The difference in BRAP expression between cancer and normal tissues was analyzed by combining the data for normal tissues from the GTEx database with the data from The Cancer Genome Atlas (TCGA). Level 3 RNA sequencing data and clinical follow-up information for patients of 33 types of cancers (ACC: adrenocortical carcinoma, BLCA: bladder urothelial carcinoma, BRCA: breast invasive carcinoma, CESC: cervical squamous cell carcinoma, CHOL: cholangiocarcinoma, COAD: colon adenocarcinoma, DLBC: lymphoid neoplasm diffuse large B-cell lymphoma, ESCA: esophageal carcinoma, GBM: glioblastoma multiforme, LGG: brain lower grade glioma, HNSC: head and neck squamous cell carcinoma, KICH: kidney chromophobe, KIRC: kidney renal clear cell carcinoma, KIRP: kidney renal papillary cell carcinoma, LAML: acute myeloid leukemia, LIHC: liver hepatocellular carcinoma, LUAD: lung adenocarcinoma, LUSC: lung squamous cell carcinoma, MESO: mesothelioma, OV: ovarian serous cystadenocarcinoma, PAAD: pancreatic adenocarcinoma, PCPG: pheochromocytoma and paraganglioma, PRAD: prostate adenocarcinoma, READ: rectum adenocarcinoma, SARC: sarcoma, SKCM: skin cutaneous melanoma, STAD: stomach adenocarcinoma, TGCT: testicular germ cell tumors, THCA: thyroid carcinoma, THYM: thymoma, UCEC: uterine corpus endometrial carcinoma, UCS: uterine carcinosarcoma, UVM: uveal melanoma) were obtained from TCGA database and downloaded through the NCI Genetic Data Commons (GDC^3^). All expression data were normalized through log2 conversion.

### MMR Gene Mutation and DNA Methyltransferase Analysis

The mutation levels of five mismatch repair (MMR) genes (MLH1, MSH2, MSH6, PMS2, and EPCAM) were obtained from TCGA database. Pearson correlation analysis was used to evaluate the relationship between BRAP expression and MMR gene mutation levels. DNA methyltransferase plays an important role in altering chromatin structure and gene expression. In this study, we also used Pearson correlation analysis to estimate the relationship between BRAP expression and four methyltransferases (DNMT1, DNMT2, DNMT3A, and DNMT3B).

### Prognosis Analysis

The correlation between BRAP expression and patients’ prognosis, such as overall survival (OS), disease-free interval (DFI), and progression-free interval (PFI), was analyzed in 33 types of cancers and shown by forest plots and Kaplan–Meier curves. Hazard ratio (HR) with 95% confidence intervals and log-rank *P*-value were calculated *via* univariate survival analysis.

### Immune Correlation Analysis

Tumor Immune Evaluation Resource (TIMER) is a database for systematic analysis of immune infiltration (B cells, CD4 + T cells, CD8 + T cells, neutrophils, macrophages, and dendritic cells) across diverse cancer types^4^. In this study, infiltrating immune cell scores of 33 cancer types were downloaded from the TIMER database. Spearman correlation analysis was used to evaluate the correlation between BRAP expression and the scores of these immune cells, including B cells, CD4 + T cells, CD8 + T cells, dendritic cells, macrophages, and neutrophils. In addition, the correlation between BRAP expression and immune checkpoint marker levels was explored *via* correlation modules.

### Statistical Analysis

Kruskal–Wallis test was used to analyze BRAP expression levels in different normal tissues and different tumor cell lines. Differences in BRAP expression levels in tumor tissues and normal tissues were evaluated by *t* test. Univariate survival analysis was used to analyze the correlation of BRAP expression and patients’ survival. Kaplan–Meier methods were used to compare survival by different levels of BRAP expression. Pearson correlation analysis was calculated between BRAP expression and MMR gene mutation level, methyltransferases level, and immune checkpoint marker level. Correlations were considered significant and positive when *P* < 0.05 and *r* > 0.20. *P* < 0.05 was considered significant for all statistical analyses.

## Results

### BRAP Is Abnormally Expressed in Human Pan-Cancer

**FIGURE 1:**
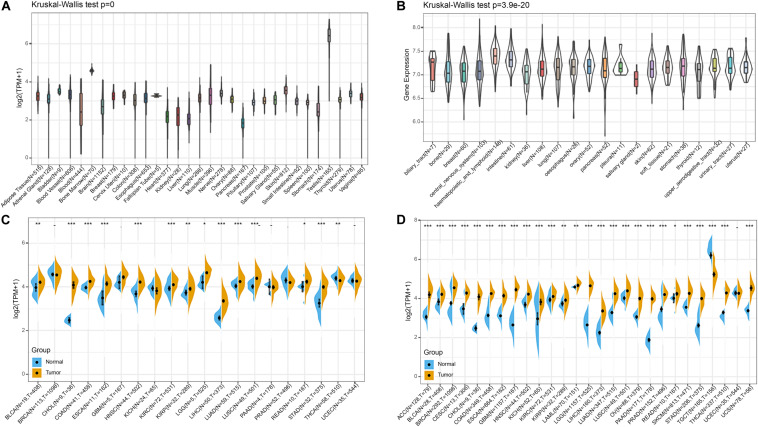
BRAP is abnormally expressed in human pan-cancer. **(A)** BRAP expression in 31 tissues from the GTEx database. **(B)** BRAP expression in 21 tumor cells from the CCLE database. **(C)** Differential expression of BRAP in cancers and normal tissues from TCGA database. **(D)** BRAP is abnormally overexpressed in 25 cancer types from the GTEx database and TCGA database (*P < 0.05, **P < 0.01, ***P < 0.001).

Firstly, we analyzed BRAP expression in 31 tissues using the GTEx database. As shown in Figure 1A, we found that BRAP was highly expressed in the testis tissue and lowly expressed in the pancreas, liver, stomach, and other normal tissues. Further, we downloaded the data of tumor cell lines from the CCLE database and analyzed BRAP expression in 21 tumor cells. As shown in Figure 1B, we found that BRAP was expressed in all 21 kinds of tumor cells. To determine the difference of BRAP expression in tumor and normal tissues, the BRAP mRNA levels in different tumors and normal tissues of 20 cancer types were analyzed using TCGA database. Results showed that BRAP mRNA levels were significantly higher in BLCA, CHOL, COAD, ESCA, HNSC, KIRC, KIRP, LGG, LIHC, LUAD, LUSC, READ, STAD, and THCA than in normal tissues (Figure 1C). Considering the small number of normal samples in TCGA, we integrated the GTEx database and TCGA database to analyze the expression difference of BRAP in 27 cancer types. We found that BRAP expression was higher in 25 tumors including ACC, BLCA, BRCA, CESC, CHOL, COAD, ESCA, GBM, HNSC, KICH, KIRC, KIRP, LAML, LGG, LIHC, LUAD, LUSC, OV, PAAD, PRAD, READ, SKCM, STAD, THCA, and UCS than in normal tissues (Figure 1D). These results indicate that BRAP is abnormally overexpressed in human pan-cancer.

### BRAP Is Correlated With MMR Gene Mutation Levels and DNA Methyltransferases in Human Pan-Cancer

**FIGURE 2:**
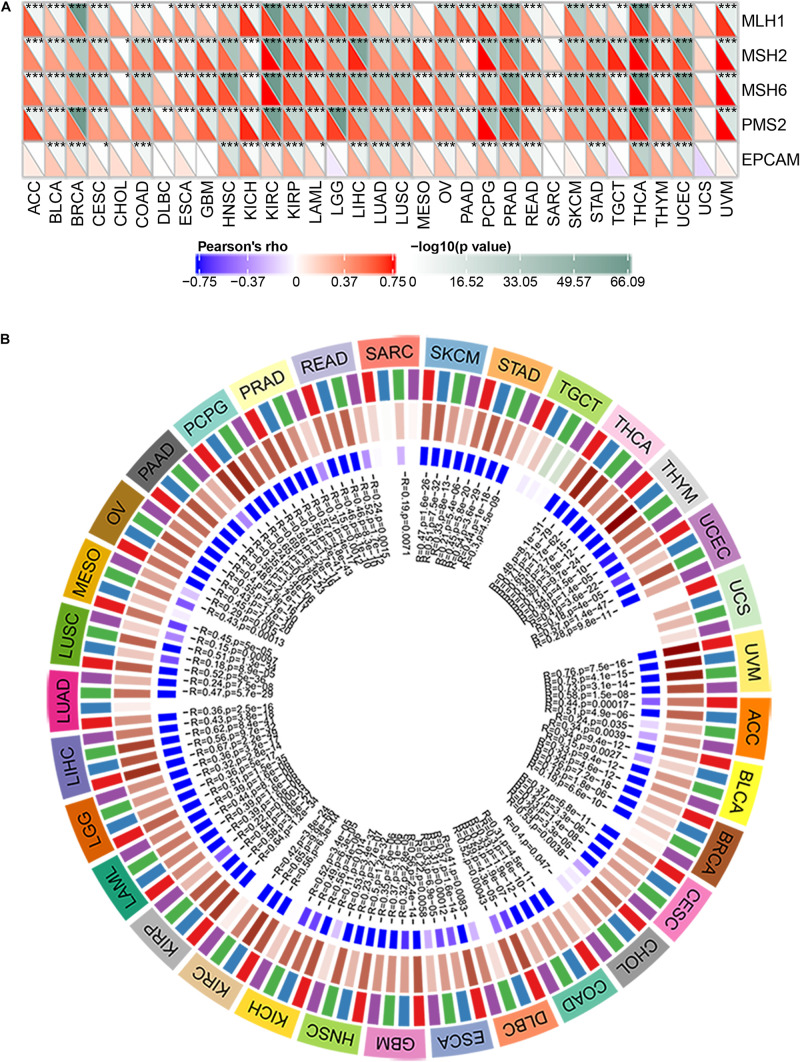
BRAP is correlated with MMR gene mutation levels and DNA methyltransferases expression in human pan-cancer. **(A)** Pearson correlation analysis of BRAP expression with mutation levels of MMR genes in human pan-cancer (*P < 0.05, **P < 0.01, ***P < 0.001). **(B)** Pearson correlation analysis of BRAP expression with DNA methyltransferases in human pan-cancer.

MMR is the mechanism of MMR in cells (Csiszar et al., 2019). The function loss of key genes in this mechanism will lead to the failure of DNA replication errors to be repaired, which will lead to the generation of higher somatic mutations and tumorigenesis (Georgakopoulos-Soares et al., 2020; McKinney et al., 2020). To assess the role of BRAP in tumorigenesis, we analyzed the correlation between BRAP expression and MMR gene mutation levels. Results showed that BRAP expression was significantly correlated with five MMR genes (MLH1, MSH2, MSH6, PMS2, and EPCAM) mutation levels in human pan-cancer and UCS was an exception (Figure 2A).

DNA methylation is an epigenetic modification that can alter gene expression (Kim et al., 2018; Masser et al., 2018; Tiffen et al., 2020). The change of DNA methylation status is an important factor of tumorigenesis (Butler et al., 2020). Therefore, we next investigated the correlation between BRAP expression and that of four DNA methyltransferases (DNMT1, DNMT2, DNMT3A, and DNMT3B) expression. Interestingly, BRAP expression was strongly correlated with DNMT1, DNMT2, DNMT3A, and DNMT3B expression in human pan-cancer, and UCS, TGCT, and SARC were exceptions (Figure 2B). In summary, there results indicate that BRAP may mediate tumorigenesis by regulating DNA damage or methylation status in human pan-cancer.

### Prognostic Potential of BRAP in Human Pan-Cancer

**FIGURE 3:**
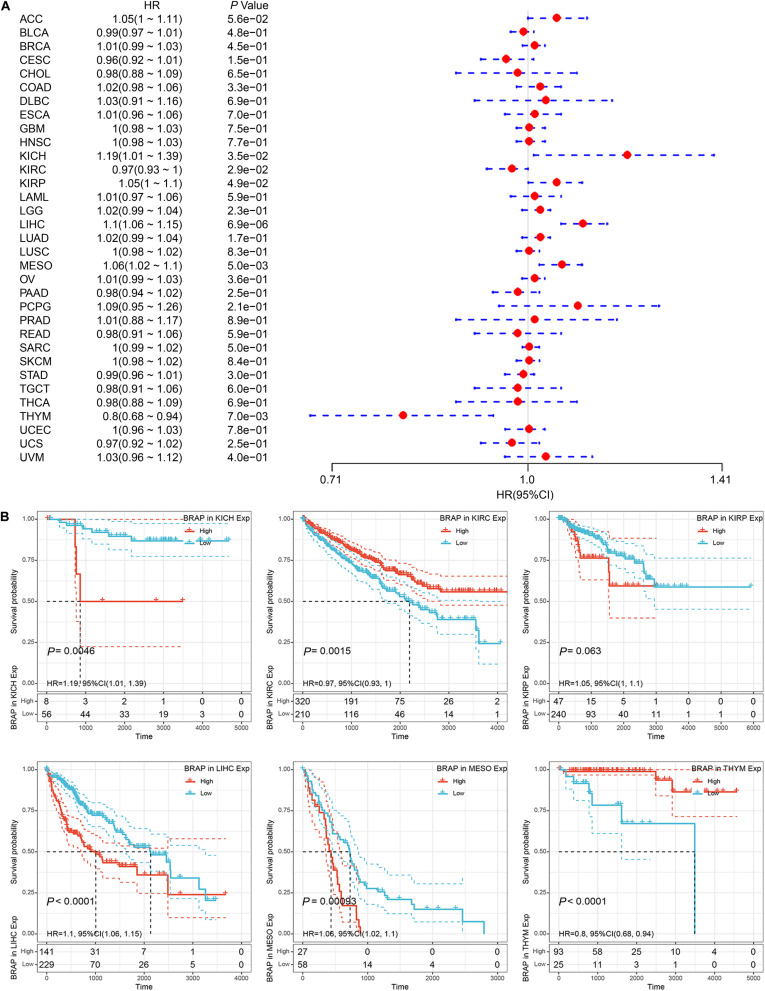
Relationship of BRAP expression with patients’ OS. **(A)** Forest plots of hazard ratios of BRAP in 33 cancer types. **(B)** Kaplan–Meier OS curves for patients stratified by different expression levels of BRAP in six cancer types.

We next investigated whether BRAP expression was correlated with the prognosis of patients in pan-cancer patients. By univariate survival analysis of 33 cancer types, we found that BRAP expression impacted patients’ OS in 6 cancer types, including KICH, KIRC, KIRP, LIHC, MESO, and THYM (Figure 3A). Moreover, Kaplan–Meier curves showed that an increased BRAP expression correlated with poor prognosis in KICH (*P* = 0.0046, HR = 1.19), KIRP (*P* = 0.063, HR = 1.05), LIHC (*P* < 0.0001, HR = 1.1), and MESO (*P* = 0.00093, HR = 1.06). KIRC (*P* = 0.0015, HR = 0.97) and THYM (*P* < 0.0001, HR = 0.8) were exceptions where high levels of BRAP showed a better prognosis (Figure 3B).

**FIGURE 4:**
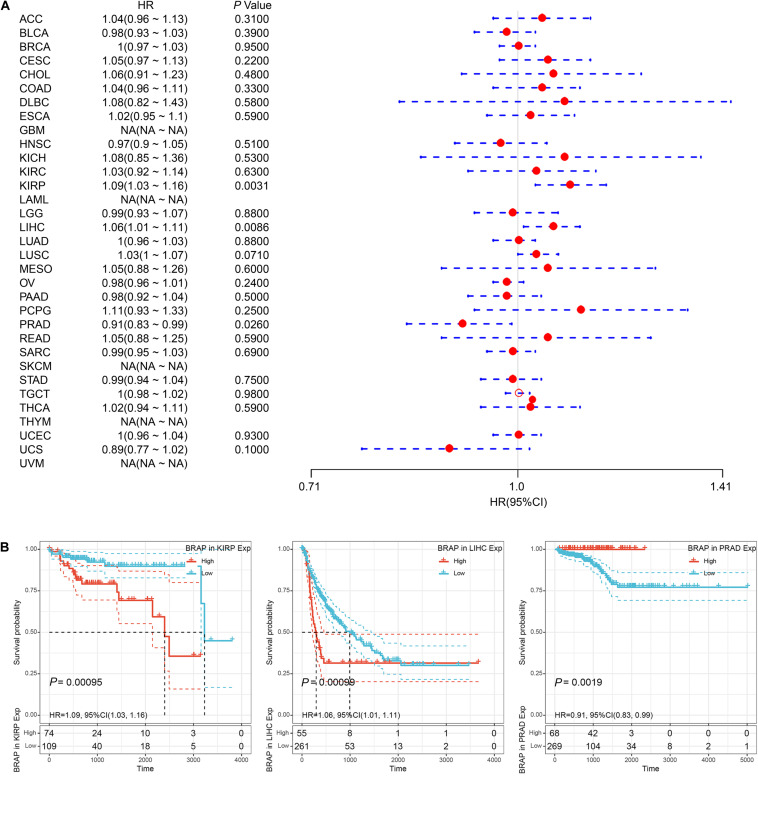
Relationship of BRAP expression with patients’ DFI. **(A)** Forest plots of hazard ratios of BRAP in 33 cancer types. **(B)** Kaplan–Meier DFI curves for patients stratified by different expression levels of BRAP in KIRP, LIHC, and PRAD.

**FIGURE 5:**
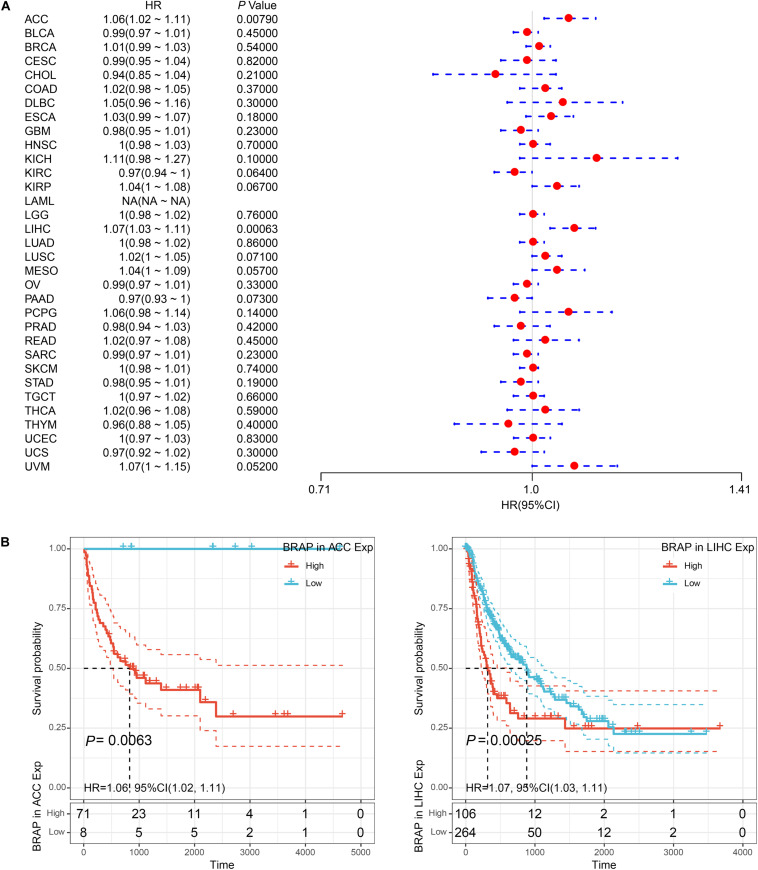
Relationship of BRAP expression with patients’ PFI. **(A)** Forest plots of hazard ratios of BRAP in 33 cancer types. **(B)** Kaplan–Meier PFI curves for patients stratified by different expression levels of BRAP in ACC and LIHC.

Furthermore, we analyzed the correlation of BRAP expression with patients’ DFI and found that BRAP expression impacted patients’ DFI in three cancer types, including KIRP, LIHC, and PRAD (Figure 4A). Kaplan–Meier DFI curves showed that an increased BRAP expression correlated with poor prognosis in KIRP (*P* = 0.00095, HR = 1.09) and LIHC (*P* = 0.00099, HR = 1.06) and reversely in PRAD (*P* = 0.0019, HR = 0.91) (Figure 4B). Meanwhile, we also analyzed the relationship between BRAP expression and PFI of patients. Forest plot revealed that BRAP expression impacted patients’ PFI in two cancer types, including ACC and LIHC (Figure 5A). Kaplan–Meier PFI curves showed that an increased BRAP expression correlated with poor prognosis in ACC (*P* = 0.0063, HR = 1.06) and LIHC (*P* = 0.00025, HR = 1.07) (Figure 5B). Overall, these results indicate that BRAP expression was strongly correlated with patients’ prognosis in many cancers, especially in LIHC, whether OS, DFI, or PFI.

### BRAP Expression Is Correlated With Immune Infiltration and Immune Checkpoint Marker

**FIGURE 6:**
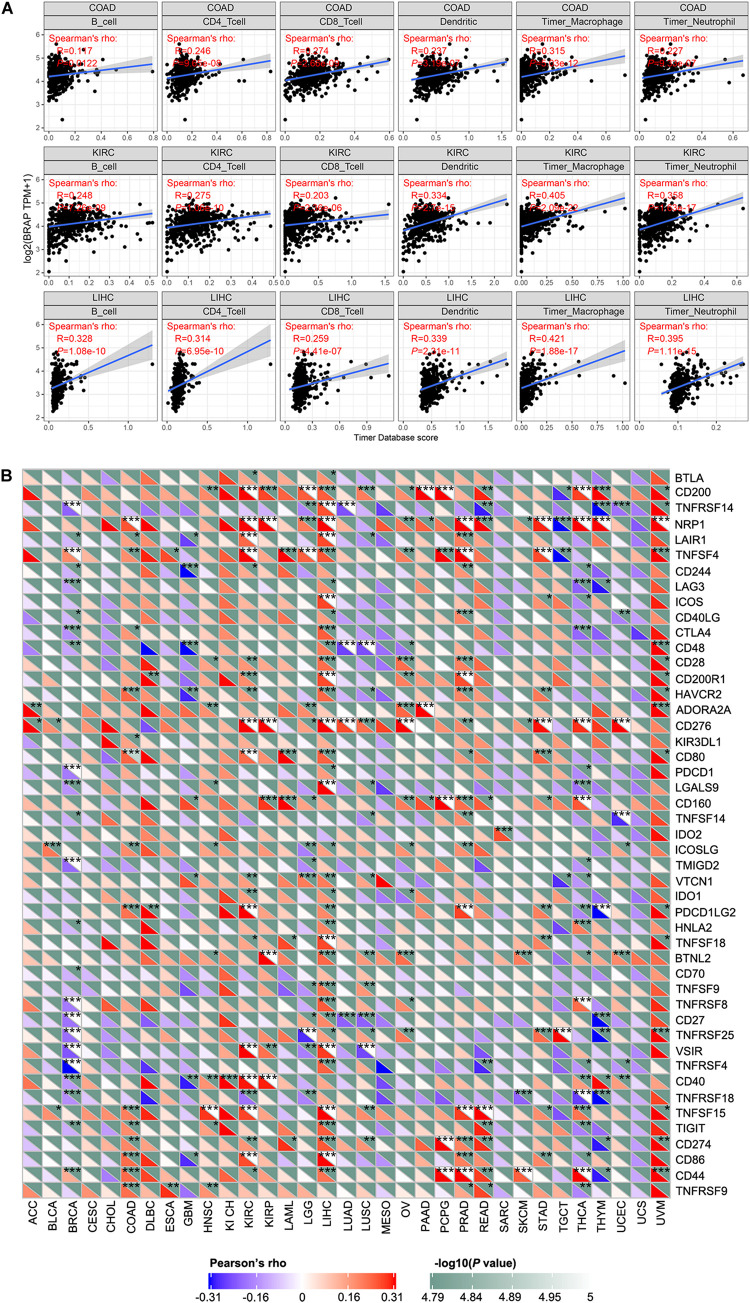
BRAP expression is correlated with immune infiltration and immune checkpoint marker. **(A)** BRAP expression is positively correlated with immune infiltration in COAD, KIRC, and LIHC. **(B)** Correlation analysis of BRAP expression level with 40 common immune checkpoint gene levels in human pan-cancer. *P < 0.05, **P < 0.01, ***P < 0.001.

Tumor-infiltrating lymphocytes are considered as independent predictors of sentinel lymph node status and patients’ prognosis in cancers (Ohtani, 2007; Azimi et al., 2012). Therefore, we studied whether BRAP expression was correlated with the level of immune infiltration in 33 cancer types from the TIMER database. Results showed that BRAP expression was significantly correlated with six types of infiltrating immune cells including B cell, CD4 + T cell, CD8 + T cell, dendritic cell, macrophage cell, and neutrophil cell in COAD, KIRC, and LIHC (Figure 6A).

Given the association of BRAP expression with immune infiltration, we next investigate the correlation between BRAP expression and immune checkpoint genes expression. We selected more than 40 common immune checkpoint genes. Interestingly, we found that in LIHC, BRAP expression was correlated with 36 immune checkpoint markers, such as CD200, TNFRSF14, TNFRSF4, CTLA4, CD28, etc. (Figure 6B). Collectively, these results suggest that BRAP plays a vital role in immune infiltration and immune escape in LIHC.

## Discussion

Pan-cancer analysis can reveal similarities and differences of tumors, which provide insights into cancer prevention and the design of therapeutic targets (Schaub et al., 2018). In recent years, many studies have focused on the pan-cancer analysis of the whole genome, revealing mutations, RNA alterations, and driver genes that are related to the occurrence and development of cancer, which is of importance for early diagnosis of cancer and development of biomarkers (ICGC/TCGA Pan-Cancer Analysis of Whole Genomes Consortium, 2020; PCAWG Transcriptome Core Group, Calabrese et al., 2020; Rodriguez-Martin et al., 2020). BRAP is an ESCC susceptible gene and identified to play vital roles in ESCC metastasis in our previous study (Zhao et al., 2017). Although BRAP has been extensively studied in ESCC, its roles in pan-cancer and whether it can be used as a biomarker are still unknown. In this study, we first comprehensively analyzed BRAP in human pan-cancer. We found that BRAP was abnormally overexpressed in 25 types of cancer and significantly correlated with MMR and DNA methylation. In addition, we found that a high expression of BRAP was associated with a poorer prognosis in LIHC, whether OS, DFI, or PFI. Furthermore, we observed that BRAP expression was positively correlated with immune infiltration and immune checkpoint markers in various types of cancer, especially in LIHC. These results strongly indicate that BRAP may be a potential biomarker and play vital roles in tumor immunity.

Previous studies showed that BRAP modulated MAPK signaling (Matheny et al., 2004). MAPK signaling pathway plays an important role in the regulation of cell growth, survival, and differentiation that are linked to the development of cancer (Lewis et al., 2018; Guo et al., 2020). In addition, genetic polymorphisms in BRAP locus are associated with a risk of colorectal cancer (Schumacher et al., 2015). Our last study showed that BRAP amplification occurs in many types of human cancer (Zhao et al., 2017). These previous studies all indicate that BRAP may be aberrantly expressed in various cancers and play important roles in cancer carcinogenesis and progression. In the present study, we found for the first time that BRAP is abnormally overexpressed in human pan-cancer including ACC, BLCA, BRCA, CESC, CHOL, COAD, ESCA, GBM, HNSC, KICH, KIRC, KIRP, LAML, LGG, LIHC, LUAD, LUSC, OV, PAAD, PRAD, READ, SKCM, STAD, THCA, and UCS compared with normal tissues. These results are consistent with our previous study that BRAP is overexpressed in a high proportion of ESCC compared with their normal counterparts.

In normal cells, MMR ensures the high fidelity of DNA replication. The MMR system is composed of several heterodimers including MLH1/PMS2, MSH2/MSH6, and EPCAM that recognize and correct gene mutations including base substitutions, insertions, deletions, or mismatches during DNA replication (Boland and Goel, 2010). Mutation or defects in MMR gene can result in increasing accumulation of genetic errors and lead to genome or microsatellite instability that attributes to the occurrence of tumors (Armaghany et al., 2012). These indicate that MMR gene mutation is a predictor of tumorigenesis. In the present study, by correlation analysis, we found that BRAP expression was strongly correlated with five MMR genes (MLH1, MSH2, MSH6, PMS2, and EPCAM) mutation levels in human pan-cancer and UCS and CHOL were exceptions. The alteration of DNA methylation is one of the important factors of tumor development (Szigeti et al., 2018). Recent research has shown that hypermethylation of the gene promoter is a common epigenetic feature of cancer (Manoochehri et al., 2020; Yin et al., 2020). In the present study, we found that BRAP expression was strongly correlated with DNMT1, DNMT2, DNMT3A, and DNMT3B expression in human pan-cancer, such as ACC, BLCA, BRCA, CESC, COAD, ESCA, GBM, HNSC, KIRP, LAML, LGG, LIHC, LUSC, OV, PAAD, PCPG, PRAD, SKCM, STAD, THCA, THYM, UCEC, and UVM. These results strongly support our conclusion that aberrantly overexpressed BRAP may play an important role in tumorigenesis by regulating MMR gene mutation level and DNA methylation.

In this study, we analyzed the correlation between BRAP levels and prognosis of patients in human pan-cancer. Results revealed that an increased BRAP expression correlated with poor prognosis in several tumor types, especially in LIHC. Whether OS, DFI, or PFI analysis, the survival time of LIHC patients with high BRAP expression was less than that of LIHC patients with low BRAP expression. These results indicate that BRAP may be a potential prognosis marker in LIHC.

In recent years, tumor microenvironment is a hot topic in tumor research. The immune microenvironment composed of tumor-infiltrating lymphocytes (B cells and T cells) and other immune cells (dendritic cells, macrophages, and neutrophils) is an important part of tumor microenvironment, which is regarded as the “seventh marker feature” of tumor (Junttila and de Sauvage, 2013). Tumor-infiltrating lymphocyte is a key component of the immune microenvironment. Studies reveal that it plays an important role in tumor immunity to inhibit or promote tumor progression (Woo et al., 2014; Berntsson et al., 2016; Carvajal-Hausdorf et al., 2017; Lianyuan et al., 2018; Xu et al., 2018). Classical dendritic cell is the first group of cells that initiate inflammatory response. It can present antigens to CD4 + and CD8 + T cells and bridge the natural immune and adaptive immune. Under normal conditions, it shows a strong anti-tumor immune ability. However, with the role of chemotactic factor, dendritic cells that are collected to tumor tissues are immature and impaired in antigen presentation ability, which are helpful to tumor immune escape (Srikrishna and Freeze, 2009). Immune escape is considered as one of the signs of cancer development. Macrophages constitute the first line of defense for anti-tumor immunity. However, TAMs cannot kill tumor cells, but participate in the process of tumor development (Shan et al., 2020). Neutrophils are early infiltrating inflammatory cells, which play a key role in initiating and expanding inflammatory response. Matrix metalloproteinase (MMP) secreted by neutrophils can reshape extracellular matrix, reduce the cell interaction, and enable tumor cells to escape immune surveillance and promote distant metastasis (Fetz et al., 2020; Liu and Liu, 2020). These all indicate that tumor-infiltrating immune cells play an important role in tumor progression. However, little is known about the roles that BRAP plays in the immune microenvironment. In our present study, we found that BRAP expression was significantly correlated with six immune infiltrating cells including B cell, CD4 + T cell, CD8 + T cell, dendritic cell, macrophage cell, and neutrophil cell in COAD, KIRC, and LIHC. These results are consistent with previous study that BRAP could activate inflammatory cascades (Liao et al., 2011). Moreover, the correlation between BRAP expression and immune checkpoint marker implicates the role of BRAP in regulating tumor immunology in LIHC. These novel findings constitute substantial progress in identifying the important role of BRAP in immune infiltration.

In summary, the present study has indicated that BRAP overexpression correlates with poor prognosis and increases immune infiltration levels in B cells, CD4 + T cells, CD8 + T cells, dendritic cells, macrophage cells, and neutrophil cells of multiple cancers, especially in LIHC. In addition, in LIHC, BRAP expression was strongly correlated with the immune checkpoint marker. However, these results were based on data analysis. Further experimental verification will be carried out in our next study. Therefore, BRAP may play an important role in tumor immunity and be a potential prognosis biomarker in patients with LIHC.

## Data Availability Statement

All datasets presented in this study are included in the article/supplementary material.

## Author Contributions

QJ and Y-jZ designed and supervised the study. QJ analyzed the data and wrote the original draft. X-mL and HZ edited the draft. All authors have read and approved the final manuscript.

## Conflict of Interest

The authors declare that the research was conducted in the absence of any commercial or financial relationships that could be construed as a potential conflict of interest.

## Footnotes

1 https://gtexport.org/home/

2 https://portals.broadinstitute.org/ccle/about

3 https://gdc.cancer.gov/

4 https://cistrome.shinyapps.io/timer/

## References

[B1] Albershardt T. C. Parsons A. J. Reeves R. S. Flynn P. A. Campbell D. J. Ter Meulen J. (2020). Therapeutic efficacy of pd1/pdl1 blockade in b16 melanoma is greatly enhanced by immunization with dendritic cell-targeting lentiviral vector and protein vaccine. *Vaccine* 38 3369–3377. 10.1016/j.vaccine.2020.02.034 32088020

[B2] Armaghany T. Wilson J. D. Chu Q. Mills G. (2012). Genetic alterations in colorectal cancer. *Gastrointest Cancer Res.* 5 19–27. 22574233 PMC3348713

[B3] Azimi F. Scolyer R. A. Rumcheva P. Moncrieff M. Murali R. McCarthy S. W. (2012). Tumor-infiltrating lymphocyte grade is an independent predictor of sentinel lymph node status and survival in patients with cutaneous melanoma. *J. Clin. Oncol.* 30 2678–2683. 10.1200/JCO.2011.37.8539 22711850

[B4] Berntsson J. Nodin B. Eberhard J. Micke P. Jirström K. (2016). Prognostic impact of tumour-infiltrating b cells and plasma cells in colorectal cancer. *Int. J. Cancer.* 139 1129–1139. 10.1002/ijc.30138 27074317

[B5] Bocchialini G. Lagrasta C. Madeddu D. Mazzaschi G. Marturano D. Sogni F. (2020). Spatial architecture of tumour-infiltrating lymphocytes as a prognostic parameter in resected non-small-cell lung cancer. *Eur. J. Cardiothorac. Surg.* 58 619–628. 10.1093/ejcts/ezaa098 32267920

[B6] Boland C. R. Goel A. (2010). Microsatellite instability in colorectal cancer. *Gastroenterology* 138 2073–2087. 10.1053/j.gastro.2009.12.064 20420947 PMC3037515

[B7] Butler M. Pongor L. Su Y. T. Xi L. Raffeld M. Quezado M. (2020). Mgmt status as a clinical biomarker in glioblastoma. *Trends Cancer.* 6 380–391. 10.1016/j.trecan.2020.02.010 32348734 PMC7315323

[B8] Carvajal-Hausdorf D. E. Mani N. Velcheti V. Schalper K. A. Rimm D. L. (2017). Objective measurement and clinical significance of ido1 protein in hormone receptor-positive breast cancer. *J. Immunother. Cancer.* 5:81. 10.1186/s40425-017-0285-7 29037255 PMC5644103

[B9] Cheng Y. Wang T. Lv X. Li R. Yuan L. Shen J. (2020). Detection of pd-l1 expression and its clinical significance in circulating tumor cells from patients with non-small-cell lung cancer. *Cancer Manag Res.* 12 2069–2078. 10.2147/CMAR.S245425 32256114 PMC7093656

[B10] Csiszar A. Balasubramanian P. Tarantini S. Yabluchanskiy A. Zhang X. A. Springo Z. (2019). Chemically induced carcinogenesis in rodent models of aging: assessing organismal resilience to genotoxic stressors in geroscience research. *Geroscience* 41 209–227. 10.1007/s11357-019-00064-4 31037472 PMC6544731

[B11] Cuevas L. M. Daud A. I. (2018). Immunotherapy for melanoma. *Semin. Cutan. Med. Surg.* 37 127–131. 10.12788/j.sder.2018.028 30040090

[B12] Eguren-Santamaria I. Sanmamed M. F. Goldberg S. B. Kluger H. M. Idoate M. A. Lu B. Y. (2020). Pd-1/pd-l1 blockers in nsclc brain metastases: challenging paradigms and clinical practice. *Clin. Cancer Res.* 26 4186–4197. 10.1158/1078-0432.CCR-20-0798 32354698

[B13] Fetz A. E. Radic M. Z. Bowlin G. (2020). Neutrophils in biomaterial-guided tissue regeneration: matrix reprogramming for angiogenesis. *Tissue Eng. Part B Rev.* 10.1089/ten.TEB.2020.0028 Online ahead of print 32299302

[B14] Georgakopoulos-Soares I. Koh G. Momen S. E. Jiricny J. Hemberg M. Nik-Zainal S. (2020). Transcription-coupled repair and mismatch repair contribute towards preserving genome integrity at mononucleotide repeat tracts. *Nat. Commun.* 11:1980. 10.1038/s41467-020-15901-w 32332764 PMC7181645

[B15] Gonzalo S. Coll-Bonfill N. (2019). Genomic instability and innate immune responses to self-DNA in progeria. *Geroscience* 41 255–266. 10.1007/s11357-019-00082-2 31280482 PMC6702534

[B16] Guo Y. J. Pan W. W. Liu S. B. Shen Z. F. Xu Y. Hu L. L. (2020). Erk/mapk signalling pathway and tumorigenesis. *Exp. Ther. Med.* 19 1997–2007. 10.3892/etm.2020.8454 32104259 PMC7027163

[B17] He T. F. Yost S. E. Frankel P. H. Dagis A. Cao Y. Wang R. (2020). Multi-panel immunofluorescence analysis of tumor infiltrating lymphocytes in triple negative breast cancer: evolution of tumor immune profiles and patient prognosis. *PLoS One* 15:e0229955. 10.1371/journal.pone.0229955 32150594 PMC7062237

[B18] ICGC/TCGA Pan-Cancer Analysis of Whole Genomes Consortium (2020). Pan-cancer analysis of whole genomes. *Nature* 578 82–93. 10.1038/s41586-020-1969-6 32025007 PMC7025898

[B19] Junttila M. R. de Sauvage F. J. (2013). Influence of tumour micro-environment heterogeneity on therapeutic response. *Nature* 501 346–354. 10.1038/nature12626 24048067

[B20] Kim S. Wyckoff J. Morris A. T. Succop A. Avery A. Duncan G. E. (2018). DNA methylation associated with healthy aging of elderly twins. *Geroscience* 40 469–484. 10.1007/s11357-018-0040-0 30136078 PMC6294724

[B21] Lewis K. N. Rubinstein N. D. Buffenstein R. (2018). A window into extreme longevity; the circulating metabolomic signature of the naked mole-rat, a mammal that shows negligible senescence. *Geroscience* 40 105–121. 10.1007/s11357-018-0014-2 29679203 PMC5964061

[B22] Lianyuan T. Dianrong X. Chunhui Y. Zhaolai M. Bin J. (2018). The predictive value and role of stromal tumor-infiltrating lymphocytes in pancreatic ductal adenocarcinoma (pdac). *Cancer Biol. Ther.* 19 296–305. 10.1080/15384047.2017.1416932 29313457 PMC5902243

[B23] Liao Y. C. Wang Y. S. Guo Y. C. Ozaki K. Tanaka T. Lin H. F. (2011). Brap activates inflammatory cascades and increases the risk for carotid atherosclerosis. *Mol. Med.* 17 1065–1074. 10.2119/molmed.2011.00043 21670849 PMC3188876

[B24] Liu Y. Liu L. (2020). The pro-tumor effect and the anti-tumor effect of neutrophils extracellular traps. *Biosci. Trends.* 13 469–475. 10.5582/bst.2019.01326 31866615

[B25] Manoochehri M. Wu Y. Giese N. A. Strobel O. Kutschmann S. Haller F. (2020). Sst gene hypermethylation acts as a pan-cancer marker for pancreatic ductal adenocarcinoma and multiple other tumors: toward its use for blood-based diagnosis. *Mol. Oncol.* 14 1252–1267. 10.1002/1878-0261.12684 32243066 PMC7266283

[B26] Masser D. R. Hadad N. Porter H. Stout M. B. Unnikrishnan A. Stanford D. R. (2018). Analysis of DNA modifications in aging research. *Geroscience* 40 11–29. 10.1007/s11357-018-0005-3 29327208 PMC5832665

[B27] Matheny S. A. Chen C. Kortum R. L. Razidlo G. L. Lewis R. E. White M. A. (2004). Ras regulates assembly of mitogenic signalling complexes through the effector protein imp. *Nature* 427 256–260. 10.1038/nature02237 14724641

[B28] McKinney J. A. Wang G. Mukherjee A. Christensen L. Subramanian S. H. S. Zhao J. (2020). Distinct DNA repair pathways cause genomic instability at alternative DNA structures. *Nat. Commun.* 11:236. 10.1038/s41467-019-13878-9 31932649 PMC6957503

[B29] Ohtani H. (2007). Focus on tils: prognostic significance of tumor infiltrating lymphocytes in human colorectal cancer. *Cancer Immun.* 7:4. PMC2935759 17311363

[B30] PCAWG Transcriptome Core Group, Calabrese C. Davidson N. R. Demircloglu D. Fonseca N. A. He Y. (2020). Genomic basis for RNA alterations in cancer. *Nature* 578 129–136. 10.1038/s41586-020-1970-0 32025019 PMC7054216

[B31] Rodriguez-Martin B. Alvarez E. G. Baez-Ortega A. Zamora J. Supek F. Demeulemeester J. (2020). Pan-cancer analysis of whole genomes identifies driver rearrangements promoted by LINE-1 retrotransposition. *Nat. Genet.* 52 306–319. 10.1038/s41588-019-0562-0 32024998 PMC7058536

[B32] Rožman P. (2018). The potential of non-myeloablative heterochronous autologous hematopoietic stem cell transplantation for extending a healthy life span. *Geroscience* 40 221–242. 10.1007/s11357-018-0027-x 29948868 PMC6060192

[B33] Schaub F. X. Dhankani V. Berger A. C. Trivedi M. Richardson A. B. Shaw R. (2018). Pan-cancer Alterations of the MYC Oncogene and Its Proximal Network across the Cancer Genome Atlas. *Cell Syst.* 6 282.e2–300.e2. 10.1016/j.cels.2018.03.003 29596783 PMC5892207

[B34] Schirosi L. Saponaro C. Giotta F. Popescu O. Pastena M. I. Scarpi E. (2020). Tumor infiltrating lymphocytes and nherf1 impact on prognosis of breast cancer patients. *Transl. Oncol.* 13 186–192. 10.1016/j.tranon.2019.10.020 31865181 PMC6931214

[B35] Schumacher F. R. Schmit S. L. Jiao S. Edlund C. K. Wang H. Zhang B. (2015). Genome-wide association study of colorectal cancer identifies six new susceptibility loci. *Nat. Commun.* 6:7138. 10.1038/ncomms8138 26151821 PMC4967357

[B36] Shan X. Zhang C. Wang Z. Wang K. Wang J. Qiu X. (2020). Prognostic value of a nine-gene signature in glioma patients based on tumor-associated macrophages expression profiling. *Clin. Immunol.* 216:108430. 10.1016/j.clim.2020.108430 32325251

[B37] Srikrishna G. Freeze H. H. (2009). Endogenous damage-associated molecular pattern molecules at the crossroads of inflammation and cancer. *Neoplasia* 11 615–628. 10.1593/neo.09284 19568407 PMC2697348

[B38] Stenzel P. J. Schindeldecker M. Tagscherer K. E. Foersch S. Herpel E. Hohenfellner M. (2020). Prognostic and predictive value of tumor-infiltrating leukocytes and of immune checkpoint molecules pd1 and pdl1 in clear cell renal cell carcinoma. *Transl. Oncol.* 13 336–345. 10.1016/j.tranon.2019.11.002 31881506 PMC7031108

[B39] Szigeti K. A. Galamb O. Kalmár A. Barták B. K. Nagy Z. B. Márkus E. (2018). [role and alterations of DNA methylation during the aging and cancer]. *Orv. Hetil.* 159 3–15. 10.1556/650.2018.30927 29291647

[B40] Tiffen J. Gallagher S. Filipp F. Gunatilake D. Al Emran A. Cullinane C. (2020). Ezh2 cooperates with DNA methylation to downregulate key tumour suppressors and interferon gene signatures in melanoma. *J Invest Dermatol.* 10.1016/j.jid.2020.02.042 Online ahead of print 32360600

[B41] Walker E. M. Slisarenko N. Gerrets G. L. Kissinger P. J. Didier E. S. Kuroda M. J. (2019). Inflammaging phenotype in rhesus macaques is associated with a decline in epithelial barrier-protective functions and increased pro-inflammatory function in CD161-expressing cells. *Geroscience* 41 739–757. 10.1007/s11357-019-00099-7 31713098 PMC6925095

[B42] Washah H. N. Salifu E. Y. Soremekun O. Elrashedy A. A. Munsamy G. Olotu F. A. (2020). Integrating bioinformatics strategies in cancer immunotherapy: current and future perspectives. *Comb. Chem. High. Throughput. Screen* 10.2174/1386207323666200427113734 Online ahead of print 32338212

[B43] Woo J. R. Liss M. A. Muldong M. T. Palazzi K. Strasner A. Ammirante M. (2014). Tumor infiltrating b-cells are increased in prostate cancer tissue. *J. Transl. Med.* 12:30. 10.1186/1479-5876-12-30 24475900 PMC3914187

[B44] Wu C. Hu Z. He Z. Jia W. Wang F. Zhou Y. (2011). Genome-wide association study identifies three new susceptibility loci for esophageal squamous-cell carcinoma in chinese populations. *Nat. Genet.* 43 679–684. 10.1038/ng.849 21642993

[B45] Xu Y. Lan S. Zheng Q. (2018). Prognostic significance of infiltrating immune cell subtypes in invasive ductal carcinoma of the breast. *Tumori.* 104 196–201. 10.5301/tj.5000624 28430349

[B46] Yin Y. Che K. Hu J. Hua H. Dong A. Wang J. (2020). Hypermethylation of the rsk4 promoter associated with braf v600e promotes papillary thyroid carcinoma. *Int. J. Oncol.* 56 1284–1293. 10.3892/ijo.2020.4999 32319586

[B47] Zhao Y. Wei L. Shao M. Huang X. Chang J. Zheng J. (2017). Brca1-associated protein increases invasiveness of esophageal squamous cell carcinoma. *Gastroenterology* 153 1304–1319. 10.1053/j.gastro.2017.07.042 28780075

